# The Outcomes of Dapagliflozin Use in Real-Life Clinical Settings in Endocrinology Clinics of Islamabad, Pakistan

**DOI:** 10.7759/cureus.8565

**Published:** 2020-06-11

**Authors:** Matiullah Kamin, Osama Ishtiaq, Kashif Raashid, Muhammad Umar Wahab, Sajjad Ali Khan, Umar Raja

**Affiliations:** 1 Endocrinology, Shifa International Hospital, Islamabad , PAK; 2 Endocrinology, Shifa International Hospital, Islamabad, PAK; 3 Endocrinology, Umar Diabetes and Footcare Clinic, Islamabad, PAK; 4 Diabetology, Umar Diabetes Foundation, Islamabad, PAK; 5 Endocrinology, Aga Khan University Hospital, Karachi, PAK

**Keywords:** type 2 diabetes mellitus, dapagliflozin, retrospective study, hemoglobin a1c

## Abstract

Introduction

Dapagliflozin is a member of a novel class of drugs (sodium-glucose cotransporter-2 inhibitors) used to treat type 2 diabetes mellitus and licensed in Pakistan in 2017. This retrospective observational study evaluated the effects of dapagliflozin on glycated hemoglobin (HbA1c) concentrations in patients treated at endocrinology clinics in Islamabad, Pakistan. The secondary objectives included assessing the effects of dapagliflozin on weight reduction and blood pressure control and to determining its safety.

Methodology

Patients with type 2 diabetes who were treated with dapagliflozin were identified by screening the electronic medical records at tertiary care hospitals in Islamabad. Data were collected at the first visit and at follow-up. Categorical variables were recorded as frequencies and percentages and compared by McNemar’s tests, and continuous variables were recorded as means and standard deviations and compared by paired sample t-tests.

Results

Mean HbA1C concentration was significantly lower at follow-up than at the first visit (7.57%±0.98% vs. 9.07%±2.07%, respectively; p<0.001). Bodyweight (85.09±15.92 kg vs. 87.07±16.11 kg, respectively; p<0.001) and diastolic blood pressure (80.34±7.12 mmHg vs. 82.34±9.61 mmHg, respectively; p<0.001) were also significantly lower at follow-up than at the first visit, whereas systolic pressure showed a marginally significant reduction (123.5±16.57 mmHg vs. 126.83±19.97 mmHg, p=0.048).

Conclusion

This first observational study of patients in Pakistan treated with dapagliflozin found that HbA1c concentration, weight, and blood pressure were reduced after initiation of dapagliflozin treatment.

## Introduction

Pakistan currently ranks fourth in the world in the percentage of people with diabetes mellitus, with an estimated prevalence of 26.3% [1]. Effective therapeutic agents are, therefore, needed to manage diabetes as well as to prevent diabetes-associated microvascular and macrovascular complications [2]. Sodium-glucose cotransporter-2 inhibitors (SGLT2i) are a novel class of drugs effective in managing type 2 diabetes mellitus, both as monotherapy and combined with other agents [3]. One member of this class, dapagliflozin, is a highly selective, potent SGLT2i, first licensed for use in Pakistan in 2017 [4,5].

Several studies have evaluated and confirmed the safety and efficacy of dapagliflozin in real-life clinical settings. Pakistani populations differ in genetic characteristics, as well as in demographic, cultural, and lifestyle characteristics, from the populations of Western countries [6-10]. This retrospective, real-life observational study evaluated the effectiveness and safety of dapagliflozin 10 and 5 mg once daily combined with other agents in Pakistani patients.

## Materials and methods

This retrospective observational study involved patients evaluated at endocrinology clinics of Shifa International Hospital and Umar Diabetes and Foot Care Centre in Islamabad, Pakistan. The study was conducted after approval by Ethical Review Committee. All patients with type 2 diabetes, who were treated with dapagliflozin, were identified by screening electronic medical records at these hospitals. Patients were included if they had been diagnosed with type 2 diabetes for at least six months and were treated with dapagliflozin as monotherapy or combined with one or two other oral agents or insulin. Patients were excluded if they had type 1 diabetes, a glomerular filtration rate (GFR) <45 ml / min / 1.73 m^2^, or a history of recurrent genitourinary tract infections. Patients were also excluded if they were taking this drug for weight loss, or if data were missing from their medical records.

Data were collected at the first visit and at follow-up. The date of the first prescription of dapagliflozin was defined as the medication index date. The baseline period was defined as two to three months before the medication index date. Based on available information, follow-up data were retrieved three to six months after the medication index date.

Patient information was collected using a predesigned data collection form. Demographic characteristics included age and gender, and disease-related characteristics at the first visit included duration of diabetes, number of comorbidities, microvascular complications (e.g., retinopathy based on retinal eye screening, nephropathy based on GFR below 90 ml/min/ 1.73 m^2^, neuropathy based on symptoms), dyslipidemia, and urinary albumin:creatinine ratio. Details about other antidiabetic drugs, antihypertensive agents, and lipid-lowering medications were also recorded at baseline and follow-up, as were clinical parameters, including weight, systolic blood pressure, diastolic blood pressure, glycated hemoglobin (HbA1c), and serum creatinine concentrations. For laboratory parameters, the last available measurement during follow-up (three to six months after medication index date) was compared with baseline measurements.

The safety of dapagliflozin was assessed by recording adverse effects, the proportion of patients with adverse effects, and the proportion of patients who discontinued treatment due to side effects or lack of efficacy.

Statistical analysis

All statistical analyses were performed using IBM SPSS Statistics for Windows, Version 20.0 (Armonk, NY: IBM Corp.). Categorical variables were recorded as frequencies and percentages for each category and compared by McNemar’s tests, and continuous variables were recorded as means and standard deviations and compared by paired t-tests. Predictors of post-dapagliflozin HbA1c (%) reduction and weight loss (dependent variable) were evaluated using a multivariate linear regression model. Results are expressed as odds ratios (ORs) and 95% confidence intervals (CIs). A p-value <0.05 was considered statistically significant.

## Results

**Figure 1:**
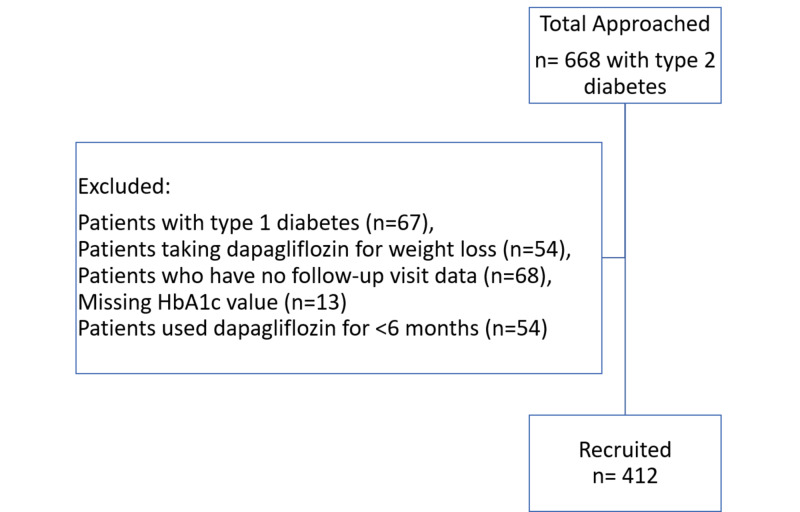
Study flow Patient flow diagram showing the profile of patients in this trial. HbA1c, glycated hemoglobin.

The study cohort consisted of 412 patients with type 2 diabetes (Figure 1). Table 1 summarizes their baseline characteristics. The 412 patients included 226 (54.9%) men and 186 (45.1%) women, with a mean age of 52.6±10.8 years and mean diabetes duration of 9.3±6.2 years. At baseline, their mean weight was 87.07±16.11 kg, and their mean HbA1c concentration was 9.07%±2.07%. At baseline, 88.3% of patients were being treated with a single antidiabetic agent, 11% were on dual therapy, and 33.7% were being treated with insulin.

**Table 1:** Baseline characteristics of study population SD, standard deviation; HbA1c, glycated hemoglobin; SBP, systolic blood pressure; DBP, diastolic blood pressure; ACE, angiotensin-converting enzyme; ARB, angiotensin receptor blocker.

Variable	Values
Age (years), mean±SD	52.6±10.8
Diabetes duration (years), mean±SD	9.34±6.20
Gender, n (%)	
Male	226 (54.9)
Female	186 (45.1)
HbA1C (%), mean±SD	9.07 (2.07)
Weight (kg), mean±SD	87.07 (16.11)
SBP (mmHg), mean±SD	126.38 (19.97)
DBP (mmHg), mean±SD	82.34 (9.61)
Comorbidity, n (%)	
No	23 (5.6)
Yes	289 (94.4)
No. of comorbidities, mean±SD	2.28±1.28
Hypertension, n (%)	212 (51.5)
Heart diseases, n (%)	47 (11.4)
Dyslipidemia, n (%)	267 (64.8)
No. of concomitant medications, mean±SD	6.33±2.39
No. of antihyperglycemic medications, mean±SD	3.07±0.99
No. of antihypertensive medications, mean±SD	1.08±1.01
Dapagliflozin dose, n (%)	
5 mg	75 (18.2)
10 mg	337 (81.8)
Antihypertensive drugs, n (%)	
ACE inhibitors	54 (13.1)
Ca-channel blockers	95 (23.1)
Diuretics	50 (12.1)
ARBs	188 (45.6)
Beta blockers	58 (14.1)
Antihyperglycemic drugs, n (%)	
None	3 (0.7)
Monotherapy	364 (88.3)
Dual therapy	45 (11.0)
Insulin	139 (33.7)
Lipid-lowering therapy, n (%)	
Statins	282 (68.4)
Simvastatin	14 (3.4)
Atorvastatin	113 (27.4)
Rosuvastatin	137 (33.3)
Pitavastatin	23 (5.6)
Other lipid-lowering medications, n (%)	
Ezetimibe	22 (5.3)
Fenofibrate	20 (4.8)

**Figure 2:**
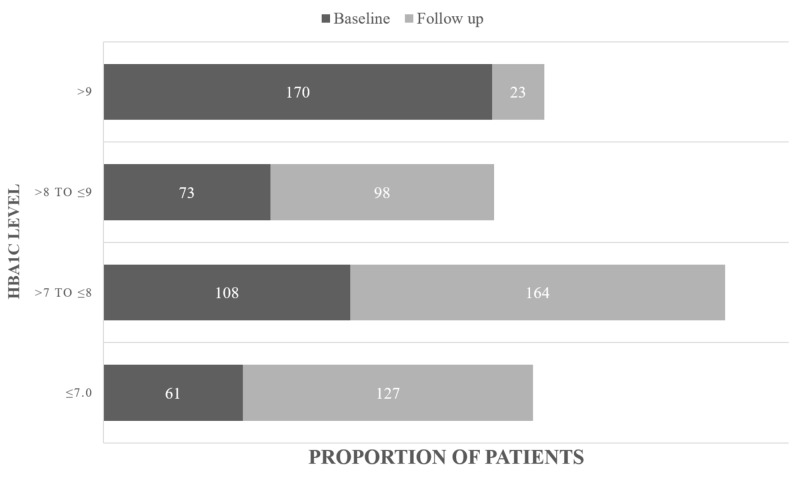
Levels of HbA1c at baseline and follow-up Numbers of patients achieving various HbA1c levels at baseline and after three to six months months of treatment with dapagliflozin. HbA1c, glycated hemoglobin.

Table 2 shows changes in mean clinical outcomes from the baseline (first visit) to follow-up in these patients. HbA1c concentration was significantly lower at follow-up than at baseline (7.97%±0.98 vs. 9.07%±2.07, respectively; p<0.001). The proportion of patients who achieved different levels of HbA1c at baseline and follow-up within six months are presented in Figure 2. Bodyweight (85.09±15.92 kg vs. 87.07±16.11 kg, p<0.001) and diastolic blood pressure (80.34±7.12 mmHg vs. 82.34±9.61 mmHg, respectively; p<0.001) were also significantly lower at follow-up than at baseline, whereas systolic pressure showed a marginally significant reduction (123.5±16.57 mmHg vs. 126.83±19.97 mmHg, respectively; p=0.048). 

**Table 2:** Changes in clinical outcomes from baseline to three to six months after starting dapagliflozin treatment in patients with type 2 diabetes HbA1c, glycated hemoglobin; SBP, systolic blood pressure; DBP, diastolic blood pressure.

Variable	Baseline	Follow-up	Change	P value
Weight (kg)	87.07±16.11	85.09±15.92	-1.98±3.03	<0.001
HbA1C (%)	9.07±2.07	7.97±0.98	-1.1±1.43	<0.001
SBP (mmHg)	126.83±19.97	123.5±16.57	-2.80±15.67	0.048
DBP (mmHg)	82.34±9.61	80.34±7.12	-2.02±7.68	<0.001

Of these patients, 43.7% reported that dapagliflozin had no adverse effects, whereas 27.3% reported some minor adverse effects, the most frequent being polyuria, urinary tract infections, and genital infections (Table 3). About 4% developed hypoglycemia, whereas 29% discontinued treatment due to a lack of efficacy.

**Table 3:** Side effects of dapagliflozin UTI, urinary tract infection.

Variable	N (%)
None	180 (43.7)
Hypoglycemia	19 (4.6)
Body spasm	2 (0.5)
Fatigue	1 (0.2)
Genital infections	4 (1.0)
Gastrointestinal problems	1 (0.2)
Headaches	1 (0.2)
Joint pains	1 (0.2)
Polyurea	11 (2.7)
Shivering	1 (0.2)
Skin rashes	2 (0.5)
Urinary problems	5 (1.2)
UTI	5 (1.2)
Weakness	1 (0.2)
No benefit	29 (7.0)

## Discussion

This retrospective analysis was the first study in Pakistan to assess the safety and efficacy of an SGLT2i in patients with diabetes mellitus. Dapagliflozin initiation was found to significantly improve clinical parameters in patients with diabetes in a real-world clinical setting, reducing HbA1c concentration, weight, and blood pressure, similar to the results of clinical trials. Of the included patients, 30.8% achieved a target HbA1c ≤7% after starting dapagliflozin. Moreover, the proportion of patients with HbA1c >9% decreased significantly, from 41.26% (n=170) at baseline to only 5.58% (n=23) at follow-up. These findings indicated that dapagliflozin-associated improvement was greater among patients with poor glycemic control, despite the study cohort being younger and having a longer duration of diabetes than other study cohorts.

One of the benefits of SGLT2 inhibitor treatment in patients with type 2 diabetes mellitus is the weight loss associated with this class of drugs, a study showed weight loss change versus placebo was -1.3 to -2.0 kg [11]. In the present study, the mean weight change after dapagliflozin initiation was -1.98 kg, comparable to findings in a large real-world cohort, which reported a mean weight change of -2.20 kg after treatment with dapagliflozin for three to six months.

Although 27% of the patients in this study experienced side effects of dapagliflozin, only a small percentage had urinary tract infections and genital infection. This may have been due to the use of wet hygiene practices in Pakistani populations, resulting in lower rates of urinary tract and genital infections than those observed in other clinical studies of dapagliflozin [12]. Also, only 4% of the participants in the present study developed hypoglycemia, similar to previous findings [13]. However, the patients in our study who developed hypoglycemia were already on insulin or a sulfonylurea, suggesting that hypoglycemia in these patients was not primarily caused by dapagliflozin.

This study had several limitations, mainly concerning the source of the data. For example, the interval between HbA1c measurements was not constant. Also, patient records did not mention whether insulin doses were reduced after they started taking dapagliflozin. Because this study analyzed patients treated with dapagliflozin for three to six months, it was not possible to predict therapeutic persistence. Large-scale real-life studies are needed to determine whether treated with SGLT2is or hygiene practices were responsible for the reduced rates of urinary tract and genital infections in Pakistani populations. An ongoing open-label randomized controlled trial is testing the safety and efficacy of another SGLT2i, empagliflozin, in a Pakistani Muslim population (SAFE-PAK Study).

## Conclusions

This retrospective, real-life observational study evaluated the effectiveness and safety of dapagliflozin in Pakistani patients. Our findings indicate that the SGLT2i dapagliflozin is an effective agent in treating patients with type 2 diabetes, with good efficacy and safety profiles. Dapagliflozin use in patients with diabetes is associated with improved glycemic control by controlled excretion of glucose through the urine and weight loss as a secondary, additional benefit. Furthermore, it had a positive impact on the cardiovascular dynamics by maintaining blood pressure. However, dapagliflozin use is associated with a mild risk of urinary tract infection, albeit lower than what has been previously reported. Dapagliflozin is a useful treatment option for patients in this cohort with type 2 diabetes.
